# French

**Published:** 1897-04

**Authors:** Alexander Glass

**Affiliations:** Philadelphia, Pa.


### FRENCH.

UNDER THE DIRECTION OF ALEXANDER GLASS, V.S.,
PHILADELPHIA, PA.

A REPORT ON THE EXPULSION OF THE ŒSTRUS EQUI LARVA (BOTS) FROM THE HORSE. Professor Perroncito, of the Veterinary College of Turin, thinks that the larva of the œstrus can be expelled from the horse by means of bisulphide of carbon administered in gelatin capsules. The antiparasitic properties of the drug have been fully demonstrated in the destruction of the phylloxerilla.

There have been two reports made on this subject by Dr. Tosegni, veterinarian to the breeding establishment of Grosseton, which are very interesting. In the first report Dr. Tosegni picked out fifteen colts whose general condition indicated the presence of the œstrus larva, which was further confirmed by the fact that they passed several of them per anum. A number of capsules, each containing 12 grammes of bisulphide of carbon, were prepared; four were given to each colt, making in all 48 grammes, and they were fed their ordinary quantity of food; on the following day they were each given a purge of castor-oil. One hour after the administration of the medicine they all had an abundant secretion of saliva and constant movements of the muscles of mastication; three of the colts showed considerable nervous excitement, and all showed more or less depression some time after the drug had been administered. Three days after the administration of the capsules the expulsion of the larva commenced. The parasites were all dead when expelled. Each colt passed numbers varying from 15 to 56, the whole number passed by the fifteen colts being 592.

A second report by the same veterinarian, in which he followed the same treatment in six colts, but from his former experience when he found all the larva dead the purgative was omitted. On the third day the larva commenced to pass from the animals, and varied in number from 3 to 105 for each animal.

Another Italian veterinarian, Dr. Bugarli, followed the bisulphide of carbon treatment in two horses that were out of condition and showed want of nutrition and several of the larvæ were passed per rectum. He administered three capsules of bisulphide of carbon each containing 10 grammes of the drug, and followed it up the next day by a purgative. One horse passed 238 and the other 23.

These observations prove the efficacy of bisulphide of carbon in expelling the œstrus larvæ from the stomach, and from the above experience Dr. Perroncito thiks that the dose should be 10 grammes for horses and 8 for colts, administered in gelatin capsules. —*Giornaledella Reale Societa ed Academia Veterinaria Italiana*, August, 1896.

---

The Cook County (Illinois) Agricultural Society strongly indorses the movement to establish a State Veterinary College, and recommends its location in Chicago, owing to the great clinical facilities offered.

